# Transition to hybrid teaching of mathematics: challenges and coping strategies of Swedish teachers

**DOI:** 10.1007/s43545-023-00680-0

**Published:** 2023-05-28

**Authors:** Elin Ottergren, Ernest Ampadu

**Affiliations:** Royal Institute of Technology, Department of Learning, Stockholm, Sweden

**Keywords:** Covid-19 pandemic, Mathematics teachers, Coping strategies, Equivalency theory, Remote teaching

## Abstract

Sweden was one of the few countries that did not close down their schools during the Covid-19 pandemic. Different hybrid teaching methods were adopted, some of which are here to stay. This qualitative study explores the challenges that Swedish mathematics teachers had transitioning from face-to-face to hybrid teaching and the coping strategies that they adopted to reduce the effect of these challenges on their practices, well-being and students’ learning experiences. The results from the study were gathered from some 51 primary and secondary mathematics teachers in Stockholm using a semi-structured questionnaire. The data were analysed with cognisance of Lazarus and Folkman’s coping theory and Simonson’s equivalence theory. The results revealed that hybrid teaching had some negative impact on teaching quality, student achievement, student health, teacher workload, and teacher-student dialogue, which underpins the Swedish school curriculum. This lack of dialogue and high-quality interaction undermines the equivalency theory principle, which suggests that the format of instruction should not influence the quality of students’ learning experiences. Also, the results revealed that majority of the teachers ascribed positively to problem-based coping strategies as compared to the emotion-based coping strategy as they worked hard to provide students with good learning opportunities.

## Introduction

After the declaration of Covid-19 as a pandemic by the World Health Organization in 2020, governments and educational authorities were forced to look for alternative ways of teaching as the usual face-to-face approach was no longer sustainable (Palau et al. 2021). A March 2020 report by European Schoolnet (2020) established that some 87.4% of students enrolled in schools across the world—that is, over 1.5 billion students in some 181 countries—had their education abruptly disrupted as a result of the pandemic. Furthermore, a UNESCO (2020a) report suggested that, as of 13 April 2020, a total of 194 countries had closed down all their schools as a result of the pandemic and the ever-increasing number of infections at that time. The provision of meaningful educational experiences for students during the pandemic and at the same time limiting the spread of the virus were the major concerns for most governments and educational authorities. However, due to the economic, resource and technological differences across countries, UNESCO advocated for the need to identify different strategies for distance education purposes through the use of high-tech, low-tech and non-technological approaches to support student learning during the pandemic (Erümit 2021).

During the transition period from the usual face-to-face to a non-face-to-face education mode, different countries adapted different distance education models for different purposes. Emergency Remote Teaching (ERT) was the common name for most of the models implemented by different schools. Hodges et al. (2020) defined ERT as a temporary shift of teaching and learning delivery to an alternative delivery mode due to crises circumstances. Keese et al. (2022) reported that remote learning emerged as an emergency protocol allowing schools to continue instruction despite the closure of physical campuses. ERT was new to many students and teachers as well as educational authorities but they had no option but to adopt it, as the majority of schools were closed down because of the pandemic and there was a need to look for innovative ways of providing learning opportunities for students at all levels of education. Sweden was one of the most affected countries in the Nordic region in terms of the number of infections and deaths as a result of the pandemic. However, while the Covid-19 pandemic disrupted most school systems around the world, Sweden, unlike other countries, did not close its schools down, especially not its primary schools.

The Swedish government-initiated ways of making sure students were not disadvantaged by keeping schools open almost throughout the period. On 19 March 2020, the government imposed a law allowing primary and middle schools to decide whether to close and conduct their education online, and only a few compulsory schools decided to close down (Skolvärlden 2020). A generalisation of the Swedish Covid strategy as far as children and adolescents are concerned could be interpreted and formulated as: *“the younger you are, the less the pandemic should affect you in your day-to-day life”*. This is how the authors understood the Swedish Public Health Agency’s general advice to the schools’ principals as well as to the general public. In general, schools for years 0–6 remained open in Sweden during the whole period from the outbreak in March 2020 until the summer of 2021. Precautions were taken as soon as a local outbreak was imminent and, in these cases, new legislation as of spring 2020 gave principals the right to close a whole school or part of a school regardless of what grade was affected. Lower secondary (years 7–9) schools were partially closed during the second half of autumn, winter and the first part of spring in the academic year 2020–2021. Many schools incorporated intermittent scheduling for their students so that not all students were on-site at the same time, with the introduction of hybrid teaching in most Swedish secondary schools.

Upper secondary schools (years 10–12) and all adult education on campuses were shut down by mid-March 2020. In less than a week, teachers at these levels made the changes necessary to implement emergency remote teaching. This then went on for most of the academic year of 20–21, with a short break during early autumn 2020. A lot of schools offered assessments on-site or incorporated intermittent scheduling. In November of 2020, the Swedish Schools Inspectorate (SSI, Skolinspektionen) in their report on how Covid-19 had impacted the Swedish school system revealed that there had been intense work in schools to cope with the situation (Skolinspektionen 2020a). The report further suggested that most principals expressed their preparedness to handle any necessary adjustments required to alleviate the negative effects of the pandemic, especially when data in schools have shown that student absenteeism was much greater than normal (Skolinspektionen 2020a, 2020b). For example, an analysis of student absenteeism by Öckert (2020) shows that it increased by nearly 70% in compulsory school settings during the spring of 2020. However, with regard to this, Skolinspektionen (2020c) also reported that the pandemic had not had that negative impact on the knowledge of younger students as compared to those in higher grades. This was echoed in recent reports from upper secondary schools that suggested that student health and achievement were negatively impacted by remote teaching (Skolinspektionen 2021). The non-closure of schools, therefore, led to the adoption of different modes of teaching for which the use of a hybrid system of teaching was the most common form in most Swedish schools.

In most Swedish schools, some students joined classes online while others joined face-to-face, especially in secondary schools. The Swedish government and the country’s ability to keep their schools open during the pandemic, has received different interpretations and criticisms at both the local and international levels. The transition to hybrid teaching has now become the underlining principle for curriculum delivery and most people are of the view that hybrid teaching has come to stay and that there is a need to integrate this into our classroom discourse. Although the importance of the integration of technology in the teaching and learning process cannot be underestimated, it is worth examining the challenges and coping strategies of mathematics teachers as they transitioned from face-to-face to hybrid teaching especially because of the unique Swedish situation as one of the few countries that kept most of their schools open during the pandemic. The purpose of this study, therefore, is to examine the challenges and coping strategies of Swedish mathematics teachers during the transition. It is anticipated that the findings from this study will provide some insight into the kind of preparations needed when trying to provide different learning opportunities for students including learning at distance or through hybrid teaching. The study is guided by the following research questions:

1. What challenges do mathematics teachers encounter during the transition to hybrid teaching?
2. What coping strategies are adopted by mathematics teachers during the transition to hybrid teaching?

## Literature review

### Distance and emergency education

Access to education is considered a basic human right and the development of the country’s human capital through education has become the surest way of providing individual and national development in many countries. The emergence of distance education dates back about 300 years; however, curriculum instruction across the globe has changed dramatically as a result of the pandemic, with many schools moving their teaching and learning activities online and some adopting a blended learning approach (Clark 2020; Sahlberg 2021). The concept of distance education has been examined from different perspectives by different authors and researchers. For example, Roblyer and Edwards (2000) described distance education as “the acquisition of knowledge and skills through mediated information and instruction, encompassing all technologies and other forms of learning at a distance” (p. 192). Hebebci et al. (2020) also described distance education as a computer-based form of education in which the interaction between students and teachers is provided from a certain location in cases where classroom education cannot be performed due to limitations. Distance education can also combine both traditional education modes with online tasks and activities in an attempt to integrate the advantages of face-to-face and web-based teaching and learning (Amir et al. 2020). However, as discussed above, during the pandemic most schools were forced to adapt to different forms of distance education, and ERT was the most common form adopted by many schools.

The concept of emergency education has been examined from different perspectives and, according to Taka (2021), emergency education refers to education for populations affected by unforeseen situations such as armed conflict or natural disasters. Education in emergencies provides immediate physical and psychosocial protection, as well as life-saving knowledge and skills (UNESCO 2015a). In another definition, by UNESCO (2015b), emergency education could be described as all forms of educational opportunities provided during a crisis created by conflicts or disasters which have destabilised or destroyed the education system of the country or place. In addition to this, Hodges et al. (2020) also defined emergency education as a temporal educational solution to an emergent problem and that the primary purpose of such measures is not to “re-create a robust educational ecosystem but rather to provide temporary access to instruction and instructional supports in a manner that is quick to set up and is reliably available during an emergency or crisis” (p. 6).

From the above discussions, it can be argued that emergency education is all forms of ad-hoc educational opportunities created for people who as a result of conflict, natural disasters or pandemics are not able to have access to education. For this study, Hodges et al.’s (2020) definition of a temporal shift of instructional delivery to an alternative mode due to crises circumstances will be the guiding definition for distance education. As highlighted above, during the pandemic, school authorities in Sweden were given the power to determine the nature of ERT that they wanted to implement in their respective schools and this gave room for different models to be practised. Some schools run a shift system where half of the students came to school for a week and stay at home the following week, and other students engaged in online learning when they were not well. With the help of different remote and distance education platforms, the majority of students have been able to continue their learning process and even complete their education. Teachers, also were able able to conduct their duties and completed their syllabi and curriculum to help these students finish their coursework. It is, therefore, not surprising that researchers such as Basilaia and Kvavadze (2020), Dhawan (2020) and Sahlberg (2021) have argued that remote or distance education has become the order of the day and it is likely that most classrooms will continue to use different online platforms to deliver education even when the pandemic is no more, as it has been seen to be more reliable, efficient, and less stressful for both the instructor and the student. Similarly, Erümit (2021) posits that “distance education is now a solution not only in compulsory situations such as the pandemic lockdown but also as a support to facilitate education processes in both face-to-face and virtual education settings” (p. 76).

### Challenges of distance education

Despite the numerous advantages associated with remote or distance teaching and learning, research has shown that the negative effect of distance education cannot be estimated. Early research on the impact of Covid-19 on students’ learning has shown various degrees of negative impacts on students learning, achievements, learning opportunities and health outcomes (e.g. Kraft et al. 2020; Kuhfeld and Tarasawa 2020; Ocak 2020). Ocak (2020) posits that remote and distance education reduces the quality of dialogue between the teacher and the student. Due to the complex nature of the classroom, it is important to structure all lessons in such a way that individual students’ autonomy is not compromised during the teaching and learning process. However, since teachers are not able to develop and sustain quality dialogue with students during instruction sessions, it is quite difficult to provide constructive feedback that will help the individual student to develop a conceptual understanding of the concepts they are learning. Earlier studies conducted before the emergence of the pandemic also revealed similar results. For example, De Paepe et al. (2018), in their analysis of teacher perceptions of effective communication, established that most teachers teaching online are struggling to get students engaged with their coursework as not all students were able to be present during each session. They further argued that, when teaching remotely using Zoom or Teams, most teachers find it difficult to know whether a student is actively engaged or not, and using breakout rooms may be challenging as the teacher may not have time to go to all the students in their breakout rooms to provide the needed support.

Keese et al. (2022), in their analysis of K-12 teachers’ experiences of teaching during Covid-19 identified five (access to devices and internet; preparation time for transition; district/school requirements and lessons; reasonable-roll-out plans; and teachers as trainers) main challenges or barriers that teachers had in their preparation and teaching remotely. They argued that most students’ inability to have access to the required devices and unstable internet connectivity affected the teaching and learning process directly and indirectly. Similarly, Anderson and Hira (2020), in their study “Loss of brick-and-mortar schooling: how elementary educators respond”, established that most teachers had limited resources for converting their lessons to online formats. Kelley (2020), in a similar vein, also argues that most science teachers were of the view that conducting experiments online was more difficult than the hands-on-laboratory lessons that the students were used to. The teachers reported that designing a laboratory lesson to be taught remotely was time-consuming and required a lot of resources which were not readily available.

The preparation time for the transition was also another major challenge as most of the measures put in place during the transition period were ad-hoc measures and this affected the quality of the training that teachers and students had before the transition. Keese et al. (2022), in their findings, also established that national, regional, district and individual school requirements were also another major challenge as teachers tried to maintain a balance of how to satisfy the different needs, requirements and aspirations of each of these agents. This is consistent with the findings from other previous research by Samioti (2021), who posits that unclear or no directives from educational authorities, lack of equipment and unreliable internet, lack of technical support, lack of control and workload issues are the common challenges that teachers face when teaching by distance. The aspirations and desires of these different agents did not only affect the teachers in their delivery but also had both a direct and an indirect impact on the curriculum requirements, as the different agents had different views regarding the kind of learning opportunities that teachers should provide for students. Keese et al. (2022) further argue that, while some teachers were asked to only provide reviews, others were asked to keep the students engaged and others were asked to introduce students to new concepts and ideas. These inconsistencies in the curriculum requirements could be a major challenge in our quest for differentiating individual students’ needs and supporting individuals to bridge their Zone of Proximal Development (ZPD).

Apart from these challenges, UNESCO’s (2020b) report also identified stress and confusion among teachers as one of the major challenges for teachers as they tried to cope with the new normal of teaching via distance and develop mechanisms for supporting student learning. Similarly*,* Zamarro et al. (2021), in their analysis of “Understanding how COVID-19 has changed teachers’ chances of remaining in the classroom”, reported that about 25% of their respondents expressed their desire to leave their jobs at the end of the school year as compared to the national average of 16% before the pandemic. The major reason for their desire to leave their job was mainly associated with stress-related and job satisfaction issues. Moreover, previous studies have found that teaching via distance can create feelings of tension, anxiety and exhaustion. For example, De Paepe et al. (2018) argued that teachers have been under immense pressure when they have to change the mode of delivery from face-to-face to online or distance mode as they have to attend different professional development courses to be abreast with the use of these new learning platforms and tools.

### Coping strategies

From the above discussions, it is clear that these challenges are inevitable as teachers try this new method of instruction, and, in their quest to support students, they have to devise mechanisms to reduce the impact on their own work and on students’ learning. To understand how mathematics teachers coped with the challenges associated with teaching during the pandemic, the authors have decided to use the Lazarus and Folkman (1984) transactional model of stress and coping theory and Simonson’s (1999) equivalence theory. Lazarus and Folkman (1984) postulate that people cope with new demands differently and defined coping as “constantly changing cognitive and behavioural efforts to manage specific external and/or internal demands that are appraised as taxing or exceeding the resources of the person” (p. 141). For this current study, it is believed that the individual mathematics teachers’ cognitive and behavioural efforts will constantly change as a function of continuous appraisal and reappraisal of their environment and how they try to adjust to these new demands.

Lazarus and Folkman (1984) identified two different coping strategies that people, and for that matter, the mathematics teacher will adopt: emotion and problem-focused strategies. They posit that, whereas problem-focused coping is aimed at solving the problem or doing something to alter the source of the problem, an emotion-focused coping strategy on the other hand aims at reducing or managing the stress associated with the problem (MacIntyre et al. 2020). Lazarus and Folkman (1984) posit that the individual’s choice of a particular coping strategy is dependent on the kind of appraisals about the challenge. They add that the individual is more likely to adopt an emotion-focused coping strategy when “there has been an appraisal that nothing can be done to modify the challenging environmental conditions and problem-focused…are more probable when such conditions are appraised as amenable to change” (p. 150). MacIntyre et al. (2020), in their paper entitled “Language teachers’ coping strategies during the Covid-19 conversion to online teaching”, established that most teachers are more likely to first adopt an emotion-focus approach to accept the situation and attempt to deal with it through a problem-focused approach using different appraisal strategies.

As teachers navigate through these challenges and try to cope and make the necessary adjustments, the question that one would like to ask is whether this could in any way affect the quality of instruction or learning that the individual student is experiencing. To provide a holistic picture of the situation (challenges and coping strategies) under discussion, the current study adopts Simonson’s (1999) equivalency theory to examine teachers’ views regarding the effect of these challenges and their coping strategies on the learning experiences of their students. Simonson’s equivalency theory is based on the assumption that “instructional experiences are essential to learning and that no student, regardless of study mode, should be forced to endure lesser instructional experiences” (p. 7). Similarly, Lapsley et al. (2008), in their research entitled “Identical really identical? An investigation of equivalency theory and online learning”, argue that the theory “creates learning experiences of equivalent value for learners regardless of the course delivery medium” (p. 2). This should be the overarching aim of every teacher, especially in this current situation where people are not sure of how the change in the format of delivering content will affect students’ learning outcomes and experiences in general. The key components of the equivalency theory are equivalency, learning experiences, appropriate applications, students and outcomes. According to Simonson (1999), even though the environments of students learning remotely and face-to-face are different, it is the learning experiences that promote effective learning and increase students’ learning outcomes. It is, therefore. imperative for this study to elicit the views of teachers to understand how the challenges and coping strategies during their distance teaching may or may not have affected the quality of students’ learning experiences.

### Transcendental phenomenology

In understanding the lived experiences of people, several approaches have been used by different authors; however, phenomenology design is well established in the literature. Marton (1986) describes phenomenography as a research approach “adapted for mapping the qualitatively different ways in which people experience, conceptualise, perceive, and understand various aspects of, and phenomena in, in the world around them” (p. 31). For the current study, aimed at examining mathematics teachers’ transition from face-to-face to online and hybrid teaching, the mathematics teacher cannot experience a phenomenon without being experienced, and people may experience the same phenomenon differently. Only when such people’s conceptions are qualitatively examined can a holistic picture of the situation be understood (Marton and Pang 2008). Marton (2000) posits that it is not acceptable to treat a phenomenon separately from those who experience it and it as “there are not two worlds: a real, object world, on the one hand, and a subjective world of mental representations, on the other hand. There is only one world, an existing one, which is experienced and understood differently by humans. It is simultaneously objective and subjective” (p. 105).

Two approaches of phenomenology are distinguishable in the literature; hermeneutic phenomenology and transactional phenomenology. Hermeneutic phenomenology is underpinned by reflective interpretations of people’s personal experiences to achieve a meaningful understanding. In contrast, transactional phenomenology focuses on the appearance of things through textual and structural descriptions of the phenomenon (Ayala 2008; Moustakas 1994; Creswell 2013). The choice of transactional phenomenology for the current study has been informed by the purpose of the study, which is aimed at providing a logical, systematic, and coherent description of the experiences of mathematics teachers as they transition from face-to-face to online and hybrid teaching (Moerer-Urdahl and Creswell 2004). Moustakas (1994) identified three critical concepts that every researcher using transactional phenomenology should pay particular attention to; *epoche, noema*, and *noesis*. Epoche can be defined as “to stay away from or abstain”, aimed at helping the researcher perceive and receive the pure essence of the phenomenon without judgements (Moustakas 1994, p. 85).

In the present study, to overcome the bias in analysing the data, the two authors deliberately reflected on their personal experiences of teaching and learning in a face-to-face and hybrid mode during the pandemic. Both authors then strived to perceive the phenomenon as if they were experiencing or seeing this for the very first time, by putting aside their biases and presumptions. In addition, the authors split the data into two after which each author read through the individual responses and coded the data. After the initial coding, the authors exchanged their codes and discussed the individual codes before the different categories were developed. The other two elements *noema* and *noesis* have been construed differently by various authors, and most researchers overlook these two essential components because of their complexity and interrelatedness. Husserl (2003), however, provides a much more precise explanation of the two concepts. He defines *noesis* as the meaning of giving part of an act and *noema* as the meaning of an act (mind and intellect). *Noema* is that phenomenon which is experienced, and *noesis* is how the phenomenon is experienced (Husserl 2003; Moustakas 1994). In the present study, the *noema* is the transition from face-to-face teaching to online and hybrid teaching, and *noesis* is the different ways teachers experienced this transition taking into consideration the various contextual factors, which can include; students’ preparedness, availability of resources; teacher preparedness, and all other related factors.

## Method

### Research design

The current study examines the experiences of mathematics teachers as they transitioned from face-to-face teaching to hybrid teaching and, as the meanings constructed from these experiences could not be empirically measured, a qualitative design was chosen. To provide a holistic picture of the situation, the researchers adopted phenomenography to guide the data collection and analysis processes. Neubauer et al. (2019) defined phenomenology as an approach to examining the essence of a phenomenon by exploring it from the perspectives of those who have experienced it. However, since the purpose of this study was to understand the meanings that mathematics teachers attribute to their experiences, transcendental phenomenography was considered appropriate. In addition, the principles underpinning transcendental phenomenography which expect the researcher to have an open mind and set aside all preconceived ideas and allow for the emergence of the actual meanings of the phenomenon, as highlighted by Creswell (2013) and Moustakas (1994), are consistent with the objectives of the study.

### Participants and instrumentation

The target population was all primary and high school mathematics teachers in Stockholm. Criterion and convenience sampling techniques were used to select the 51 mathematics teachers who took part in the study. Through the criterion sampling technique, the researchers were able to set the criteria for selecting or reaching out only to mathematics teachers, as suggested by Patton (2014). In addition to this, since it was difficult to have access to these teachers during the pandemic, the researchers through convenience sampling techniques contacted participants who were accessible during the pandemic. The teachers from the different class levels and locations (city and suburb) represented a sample from which thick and in-depth data were generated through semi-structured questionnaires (Cresswell 2014). As highlighted by Creswell (2013), open-ended questions are appropriate for a transcendental phenomenological study because they allow participants to tell their story about the phenomenon being studied. The semi-structured questionnaire used in this study had 11 questions and the first seven questions were used to elicit information from the participants regarding their background characteristics; this is depicted in Table 1 below. The other four open-ended questions were used to gather information from the participants regarding their teaching and assessment practices, challenges and coping strategies for teaching mathematics as they transitioned from face-to-face to hybrid teaching.

**Table 1:** Demographic characteristics of participants

Gender	No	Age	No
Male	16	25–29 years	2
Female	35	30–39 years	9
Total	51	40–49 years	20
		50–59 years	14
		60 years and above	6
		Total	51

School level	No	School location	No
Primary School	10	City Centre	19
High School	41	Suburb	31
Total	51	No response	1
		Total	51

### Data sources

The researchers intended to interview the teachers to understand the challenges they had faced in teaching during the pandemic; however, it was difficult to conduct such face-to-face interviews during the pandemic. Hence, the questionnaire was circulated to different teachers within the Stockholm region via email, Google docs and other online platforms from May to June 2021. A total of 51 completed questionnaires were received after the end of June. As depicted in Table 1, the responses came from teachers from different levels, backgrounds and locations. Sixteen male and 35 female mathematics teachers from 10 primary and 41 high schools completed the questionnaire. Twenty-two (43.3%) of the respondents have 11–20 years of teaching experience and some 17 (33.3%) have 1–10 years of experience, and the majority (34) of the respondents were between the ages of 40 and 59 years, with some 11 (21.6%) between the ages of 25 and 39. The data suggest that the majority of the respondents have been in the teaching profession for quite some time and might have had the opportunity to teach under different conditions and circumstances.

### Data analysis procedure

All responses were downloaded from the different platforms, reviewed, translated from Swedish to English and edited for accuracy. The first part of the questionnaire, which had the background characteristics of the participants, was analysed using descriptive statistics. For the analysis of the open-ended responses, we followed a simplified version of Moustakas’s (1994) guidelines for transcendental phenomenology research data analysis. The data organising and analysing methods used include bracketing, coding, textual and structural descriptions, and synthesis of meanings. Because both researchers were privy to the challenges of transition to hybrid teaching, we set aside any prejudgments that we may have about the issue under investigation, and this is what Moustakas (1994) classified as bracketing. Moustakas classified this process as very important if the researcher wants to gain an authentic understanding of the phenomenon.

**Fig. 1:**
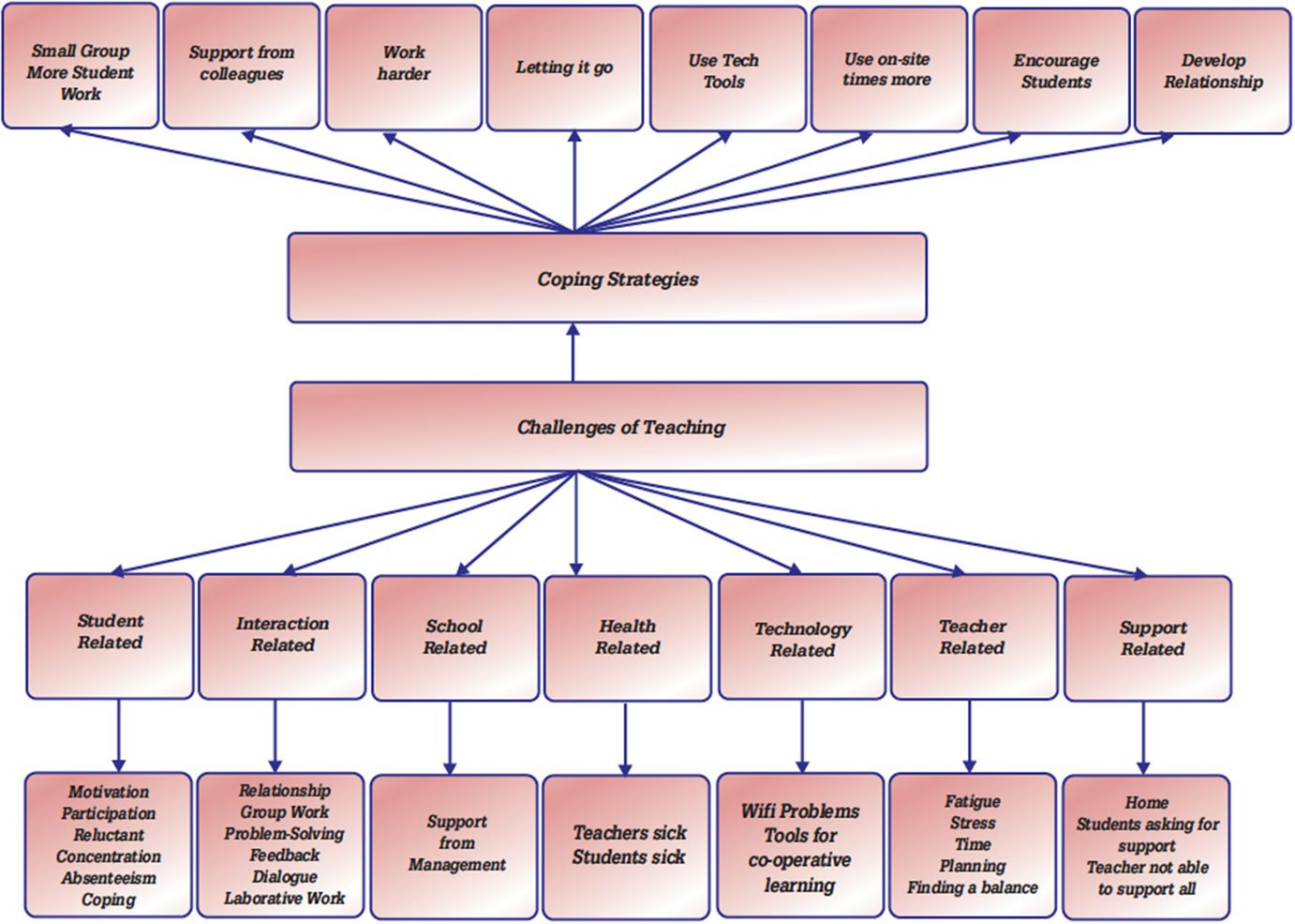
Visual presentation of themes and sub-themes

To begin with the coding process, the 51 questionnaires were divided into two (i.e., 26 and 25 questionnaires) each researcher read through the responses and first identified the different statements relevant to the topic under consideration and wrote these individual codes irrespective of how they were was indicated by the respondents. This is consistent with Moustakas’s principle of horizonalising, which suggests “every horizon or statement relevant to the topic and questions as having an equal value” (p. 118). After the coding process, both researchers came together to look at the different codes generated and started writing down textural descriptions from the different themes to help answer the questions about what the mathematics teachers experienced as they transitioned from face-to-face to hybrid teaching. In addition, structural descriptions were developed from the different themes to answer the question about how the teachers experienced the phenomenon, and this is consistent with the ideas of Creswell (2013). Finally, the combined textual and structural descriptions were used to develop the holistic picture of the experiences of the mathematics teachers as they transitioned from physical to hybrid teaching, and this is consistent with Moustakas’s synthesis of meanings and essences stage of the data analysis process. The development and identification of the different categories/themes from the responses is presented in Table 2, which was then used to develop Fig. 1.

**Table 2:** Development of categories

	Category Labels	Codes Represented	Number of Occurrences
Challenges of hybrid teaching	Student-related	Inactive students, asking for support, to be focused and motivated, students disappearing, reading students’ body language, poor response to reviews, poor progression, reluctant to group work	52
	Interaction	Getting everyone involved, difficult having dialogue, work ethics did not work	5
	School related	Enough preparation, not enough time, time consuming, more cheating during exams	12
	Health Related	Illness, teacher fatigue, not enough energy to do well	8
	Technology Related	Finding the right tools, internet connection, dependent solely on technology	5
	Teacher Related	Finding the balance, limited problem solving, lecture method, little explanation, lots of information for students to digest	8
	Support Related	Support from home, school support	4
Coping strategies during hybrid teaching	Small group and student work	Shorter reviews, small group work in breakout rooms, asking students to take pictures of their work, more individual work, more structure, few minutes of reflection	23
	Support from colleagues	Support from others, help from subject and work teams	5
	Work harder	Going to breakout rooms to provide support, developing teaching that fits, record review in advance and give to students, provide detailed solutions to the tasks, takes photos of textbooks and send to students, prep students, save discussion until students have school week	13
	Letting it go	Letting it go, lower the demands and standards, I can only do my best, making most of the time with the students who are in place at school, not waste valuable time preparing for those on distance because they won’t benefit	16
	Use tech tools	Force students to send photos of their work, exit tickets, escape rooms, used apps and websites	6
	Use on-site times more	Save discussion until students have school week, spending more time on students when they were in school	4
	Encourage students	Personal contact with students, special contacts with students, offer support in different ways	8
	Develop relationship	Developing relationships, offer support in different ways, close reconciliations with students	5

After the analysis, we identified seven themes of challenges (student, interaction, school, health, technology, teacher, and support-related challenges) and these themes/categories were derived from the 94 codes that were identified from the 51 responses to that question. In addition, eight themes were identified from the participants’ responses regarding their coping strategies (small groups, support from colleagues, working harder, letting it go, using technological tools, using on-site times more, encouraging students, and developing relationships) and these themes were also developed from the 80 individual codes that were aggregated from the 51 responses to this question. A visual representation of the different themes is presented in Fig. 1.

### Ethics considerations

The four main ethical considerations that were adhered to in this study are: confidentiality, anonymity, informed consent and voluntary participation. To achieve this, the questionnaire briefly introduced the purpose of the study, and participants were assured of the confidentiality of their responses; names of the participants, names of the school, and districts were not collected. All the names used in presenting the results are pseudonyms and do not have any traceable link to any of the participants who participated in the study.

## Results

The results of the analysis are presented with cognisance of the two research questions guiding the study.

### Research question 1: mathematics teachers’ challenges

#### Student-related factors

As highlighted above, the researchers categorised the challenges into seven different themes and a critical analysis of the results shows that all the respondents indicated that student-related challenges were the main type of challenges that affected the mathematics teachers’ teaching. Lack of motivation, participation, reluctance to learn or do assignments, concentration, absenteeism, and unable to cope with the demands of learning online were the main challenges that influenced the respondents’ teaching. This could be attributed to the fact that most of these students were not familiar with this new way of learning, and hence were not motivated to give their best as they would have done in a normal classroom situation. Such lack of motivation and enthusiasm on the part of students can have an adverse effect on the individual teacher’s teaching and the quality of his/her instruction. Some shared examples included the following:

When talking to the students, they say that it is much more difficult to stay focused and motivate themselves to work with mathematics. Many people log in to video meetings but then did other things when it’s a walkthrough and/or their work. They have lost a lot this year in my opinion (Klara, a teacher in a city secondary school).

Capturing students’ interest and learning remotely, you notice that some students find it difficult to get involved, and they are not working on the lesson. And to help students who have difficulties or have difficulty keeping up with lessons (Sara, a teacher in a suburb secondary school).

Many students have lost “school focus”. We have 7s and 8s in place every other week (at the moment) and it’s like getting students back from holidays every Monday. Their level of knowledge has dropped and many people express that they have difficulty with routines when they are at home. It’s gotten messier. Difficult to plan for the long term. It’s hard to get students with you (Katrina, a teacher in a suburban secondary school).

#### Interaction-related factors

One of the roles of the teacher in a student-centred classroom is to provide support and guidance that will help students develop a conceptual understanding of the concepts they are learning and help to bridge the individual student’s Zone of Proximal Development (ZPD). Quality of dialogue is therefore crucial in triggering critical thinking among students in the classroom. The quality of dialogue was seen as very important for most of the teachers but this was not achieved in most cases and most teachers expressed this concern as a major challenge when teaching at a distance or online, as per the following:

Getting students involved in lessons has been difficult. When they get stuck, almost no one asked for help from the classroom [when] they directly raise a hand. Students are no longer directly seen in how they are doing as they can in a classroom. There was much more micromanagement if you were going to try to get similar results. The workload for each lesson got heavier, which means that I do not have the energy to put as much energy into each lesson, but instead had to focus on me surviving myself (Lars, a teacher in a city secondary school).

It has also been much clearer that students did not seek help in the same way when working with mathematics. In a classroom, it’s easy to raise your hand or ask a companion for help, but, remotely, you should first send a message and then wait to be called. I have noticed that there is a very small percentage of students who contact [me] for help. At the joint video meetings, there are also [only] a few people who are heard asking questions, while others are completely silent (Klara, a teacher in a city secondary school).

Students became quieter and quieter as time went on. It was impossible to know until the end what they were thinking and need. I had to adjust the planning according to the individual needs of the groups. Teaching quality was difficult to maintain (Sara, a teacher in a city secondary school).

#### Health-related factors

Further reasons highlighted by some of these teachers were health-related as a result of students and teachers being infected with the coronavirus, which in turn affected teaching and learning. This was one of the concerns raised by just a few of the respondents but it is worth noting that it could have affected the quality of instruction and learning in one way or another. For example, a teacher indicated that students stay home for longer periods because they are sick and thus miss a great deal of learning; such students have to be supported and this affects planning, is time-consuming, and stressful on the part of the teacher and the student. In addition, one teacher reported that, when she was off sick for seven weeks, her students were only engaged in recreational activities, hence the students lost a lot and this also put a great deal of stress on her when she returned to work. Two respondents shared detailed examples:

Students are sick/need to stay at home for a longer period. As a result, some have “missed” larger portions of some content from the central content of the lessons. I have experienced that there is almost always some student gone or several students have gone daily. We’ve barely been the whole class at any point during this time. It’s hard to keep track of which of the students has “missed” what and hard to find the lesson time to make up for what [they have] missed (Ida, a teacher in a suburb secondary school).

Loss of knowledge and poor progression due to long-term absence [of] myself, when I was infected with COVID at work. The students didn’t really get any tuition during the little over seven weeks I was sick. They were mostly “guarded” by recreational staff during my absence. The sporadic presence among students (especially the weakest, unfortunately) combined with homes that cannot/do not want to support students in carrying out the tasks I send home (Bia, a teacher in a city secondary school).

#### Technology-related factors

Problems with WIFI or the internet and school-related factors were the least challenges mentioned by the respondents. However, a few of the participants indicated that both teachers and students had issues with internet connectivity both at home and when teachers were in school for their online sessions. Apart from connection challenges, some teachers reported that the credibility of students’ assessments has been compromised as the use of digital assessment has increased the incidence of cheating among students. In effect, teachers have been forced to ignore most of the formative assessment components and use only the summative assessment that the students do on campus, and this was detailed by one teacher:

Digital tests have increased the risk of cheating. Students use exam.net and film themselves with their mobile phones from home. A lot of technical hassle and the fact that students need to go to the toilet increases the risk of cheating. Formative assessment is difficult, it is difficult to give direct feedback to students when they work at home and you do not see what they are doing. Formative homework hearings won’t be as good. Students don’t work as well at home when they don’t have a teacher in the same room. It is especially the weak and less-motivated students who suffer from this. When the national tests were cancelled the school management did not help us organise these as before. It was a great challenge to implement this ourselves without the support of the school management (Peter, a teacher in a city secondary school).

### Research question 2: mathematics teachers’ coping strategies

The following sections provide a narrative of each theme that was developed with regard to the coping strategies that were adopted by the different participants.

#### More student work

From Table 2, it is clear that one of the coping strategies most adopted by teachers during their transition was increasing the independency of individual students where the student was given more responsibility. Out of the 74 codes that were generated and developed to form the eight categories about teachers’ coping strategies, this theme appeared 23 times where teachers described the different strategies used to force or encourage students to do more work, as evident from the following quotation:

Tried to “force” them to send in photos of what they did during the lesson. But then still some ignored it. Some had been allowed to come to school and work, just to make sure they get out of bed and get something done. But then you have to support 25 people who are sitting at home, at the same time as you have five people with a great need for support on site ... enormously stressful and an impossible task (Sara, a teacher in a suburban school).

Similarly, Elin, a teacher in a school in the city, reported that: *"I record reviews in advance, it gives a great advantage as the students can choose when they want to see the review and they can see it several times. I have also made detailed solutions to the tasks the students are expected to work with.”*

#### Letting it go

Letting it go was the second theme that a substantial number of the teachers credited as a means of managing and coping with the challenges. This theme appeared 16 times, with these teachers expressing their frustrations and how they were overwhelmed with the stress associated with teaching online and had to result to an emotion-focused coping strategy where they tried to reduce and managed the stress associated with the problem by letting it go. For example, Peter (a teacher in a city secondary school) indicated that:

You try to find ways, but it’s never as good as when you have them [students] in the classroom. Realising it and letting go of the stress of surviving yourself is boring but necessary if I’m going to last. Distance learning is neither sustainable for students nor teachers.

Similarly, while most teachers felt they had done their best, this was not reflected in students’ performance and achievement and this was evident in the responses from some of these teachers. For example, Klara (a secondary school teacher in the city) reported that:

You just do it. But I’m very tired and exhausted right now. I felt like the worst teacher in the world when the results plummet. Although I believe that what I do during their remote [learning] is of a very high level.

A report from Peter (a secondary school teacher in the suburbs) also explains his frustration and how students’ learning experiences have been influenced by distance or remote learning:

I have done quite well as I find it easy to learn digital ways of working. However, the level of stress has increased because of all the uncertainty about when we will be allowed to return to normal, whether we need to have digital tests, whether we are allowed to have national tests, etc. The teaching works well from a teacher’s perspective, but it is the examinations that are the challenge. From a student perspective, however, the teaching is not as good as when they were in their house.

In addition to this, some teachers indicated that the quality of student learning during online lessons has been far below expectations and that they had even stopped spending valuable time and energy planning distance education, and this is evidenced by the quotation from Anna (a primary school teacher in the suburbs):

Tried to make the most of the time with the students who were present at the school, so the student who were able to come to school made a lot of progress than those who joined online. I stopped spending valuable time and energy planning distance education for those who were absent because they still did not benefit from it.

#### Working hard

In situations like this, one would expect the individual teacher to make a deliberate attempt to ensure that the learning opportunities provided for students are of a high standard and that students can develop conceptual understanding irrespective of the mode of delivery. The adaptation of different strategies by these teachers was evident in their responses as they worked to provide support for their students. Working hard was the third category with the highest occurrences and the teachers described the different ways they worked hard to cope with the challenge of distance learning. Below are some quotations explaining how this was done:

I used apps and websites to try to find the same central content as what we had worked with at school so that students who were at home could have a chance to work with what we had done in school. I took pictures from the textbook so that the students who were at home would be given tasks to work with from home (Tomas, a teacher in a suburban secondary school)*.*

Before the course started this academic year, we introduced a calculation cabin in the last weeks at the school because the Math Centre was not available during the pandemic. There have been an incredible number of students who have come, so we have run several parallel groups. MA teachers were given (or rather required to receive) drawing plates because it was required to have a relatively functioning teaching. I have tried to deal with it. To summarise: extreme structure, clear communication in advance, frequent reconciliations with students, and special contact with students at risk of losing maths (Katrina, a teacher in a suburban secondary school).

## Discussion

Analysis of the data revealed that teachers’ coping strategies are consistent with Lazarus and Folkman’s (1984) identified strategies of emotion-focused and problem-focused. The majority of the teachers used problem-focused coping strategies such as small groups, more student work, support from colleagues, working harder, using technological tools, using on-site times more, encouraging students and developing relationships. The above results show that, despite teachers’ efforts in trying to implement Simonson’s equivalency theory, thereby trying to ensure that students were not forced to endure lower-quality instructional experiences, the challenges discussed above limited their efforts. The majority of students had experienced lower-quality instruction and time with their teachers, and this was reported by a majority of the respondents. In addition, some teachers indicated that they spent much more quality time with students when they attended on-site lessons as they were not able to have that kind of interaction when the students were online. As highlighted above, school principals were allowed to choose the mode of instruction that worked for them with cognisance of the government guidelines for minimising the spread of the virus. Most schools were running a hybrid approach where some students came on-site and others joined the class remotely.

Analysis of the results from the study suggests that teachers in primary school experienced few challenges pertaining to the pandemic other than the fact that students were at home to a greater extent—even if their schools were open. Absenteeism was a major challenge seen at all levels, which was to be expected due to the recommendation that students should stay home if they experienced any—even mild—symptoms. However, the results from upper secondary teachers revealed that student–student and teacher-student interaction was a major challenge. Many teachers experienced that their students had trouble staying focused or motivated during class and they often failed to complete their assignments. Almost all the secondary school respondents also mentioned that the lack of dialogue and student engagement had a very negative impact on their students’ learning. These findings were consistent with Ocak (2020), who posits that developing relationships and engaging students in high-quality dialogues were virtually absent in most classrooms as many students were not willing to talk or ask for support during the online sessions. This could be described as a major setback as “reasoning” and “communication” are two of the seven central abilities that a mathematics student must obtain, as stated in the Swedish school curriculum. The lack of dialogue in the virtual classroom is a crucial setback that may have serious negative consequences for the learners. This is consistent with the assertion of Venton and Pompano (2021) that active learning is far more successful at engaging adult STEM students than traditional lectures delivered online.

Other experiences among mathematics teachers in secondary schools included their students’ inability to meet the demands of learning online. These teachers also expressed extremely high levels of stress and feelings of inadequacy in their teaching. Similar to the findings of MacIntyre et al. (2020), most of the respondents indicated that they applied both emotion- (e.g. accepting the situation or the challenge) and problem- (e.g. strategies such as letting the students work in small groups, seeking support from colleagues or working harder) focused strategies to cope with the different challenges. Apart from the efforts from the teachers to support students, many schools made arrangements to provide on-site times to encourage students and develop teacher-learner relationships on these occasions. Despite teachers’ efforts in trying to cope with the challenges associated with teaching by distance and making sure that standards were not affected, the underlining principles of the equivalency theory were put to the test. A critical review of the results shows that the quality of instruction that students received during the pandemic is nowhere near what they experienced before the pandemic, and this was evident in the teachers’ detailed responses. This is therefore a wake-up call to all agents in the education sector as they strive to hold on to the high-quality standards of teaching and learning in Swedish schools, no matter the effect of the pandemic and the mode of delivery of instruction. A critical analysis of the results suggests that, despite the important role of technology and the fact that hybrid teaching can be one of the means of providing equal learning opportunities for all students, the challenges highlighted by these teachers cannot be underestimated.

## Implications for further research

Sustainable development goal 4 (ensure inclusive and equitable quality education and promote lifelong learning opportunities for all) underpins the background and objectives of the present study. The achievement of SDG4 has been tested in many ways since the emergence of the pandemic and, as highlighted above, providing equal learning opportunities for all students during the pandemic and after has been an issue of concern. It was therefore imperative to examine the challenges that teachers faced during the pandemic and their coping strategies. There is an overwhelming amount of research that points to a correlation between education and the well-being of citizens. It can be assumed that leaders and policymakers around the world were aware of the risks for young children, especially in socio-economically vulnerable areas, when closing down schools, and that the emergency response to do so was not a decision taken lightly. During the first quarter of 2020, many countries closed down their schools at all levels, but Swedish leaders and policymakers were not willing to do so. The Public Health Care Agency of Sweden (FoHM) emphasised the known risks for children if schools were shut down and the Swedish government followed their advice to keep schools open for as long as possible. However, by mid-March, the spread of infection became severe and, reluctantly, Sweden’s leaders, supported by the FoHM, decided to switch to remote teaching in upper secondary schools. In the autumn of 2020, the rest of the secondary schools were partially closed as well. This placed tremendously complex demands on teachers and learners, just as in other parts of the world.

## Conclusions

The emergency response to switch to remote teaching during the pandemic came with a series of adaptation strategies among school authorities, teachers and students with the hope of providing students with quality learning opportunities despite the numerous challenges that the emergence of Covid-19 has had on learners all over the world. The uniqueness of the Swedish strategy of providing learning opportunities for students during the pandemic has been critically analysed by many from different perspectives. Apart from the numerous challenges that teachers had in teaching via distance learning, the data also suggest that teachers increased their planning, gave clearer instructions and, in general offered more support for student autonomy during remote teaching periods. However, despite their efforts to ensure students had quality learning opportunities, the quality of student–student and teacher-student dialogues decreased during these periods and many teachers reported that student achievement was negatively impacted. As schools, teachers, students and educators prepare to go back to normal face-to-face instruction, it is likely that remote and hybrid learning will be incorporated into the teaching–learning processes in many schools as alternative means of providing additional learning opportunities for students. As suggested by Sahlberg (2021) and Erümit (2021), remote or distance education has become the order of the day and most classrooms will likely continue to use different online platforms to deliver education. This calls for immediate actions by governments, educational authorities, schools and teachers to harness different mechanisms to remedy the challenges discussed above. Working on finding remedies to these problems will not only help improve the quality of instruction and students’ learning outcomes, but will also go a long way to enhance teacher retention so as not to experience what was reported by Zamarro et al. (2021): that over 25% of teachers reported their desire to leave their jobs at the end of the school year as a result of these challenges.

## Data Availability

The datasets generated during and/or analysed during the current study are available from the corresponding author on reasonable request. Participants completed an online open-ended questionnaire.
